# Effectiveness and Safety of Lianhua Qingwen Capsules for COVID-19: A Propensity-Score Matched Cohort Study

**DOI:** 10.1155/2023/6028554

**Published:** 2023-02-17

**Authors:** Yun Lu, Meng Zhang, Qing-qing Yang, Wen-jing Li, Kun Yang, Wen Hu, Su-yu Gao, Qiao-li Jiang, Li-kai Lin, Hong Cheng, Feng Sun

**Affiliations:** ^1^Department of Pharmacy, Zhongnan Hospital of Wuhan University, Wuhan 430071, China; ^2^Department of Epidemiology and Biostatistics, School of Public Health, Peking University, Beijing 100191, China; ^3^Institute of Hospital Management, Wuhan University, Wuhan 430071, China

## Abstract

As a traditional Chinese medicine, Lianhua Qingwen capsules have been widely used to treat Coronavirus Disease 2019 (COVID-19). This study was aimed to demonstrate the association between treatment with Lianhua Qingwen capsules and the clinical outcomes of hospitalized patients with COVID-19. This retrospective study was conducted at four hospitals in Central China. Data of hospitalized COVID-19 patients were collected between December 19, 2019 and April 26, 2020. Based on whether Lianhua Qingwen capsules were used, patients were classified into Lianhua Qingwen and non-Lianhua Qingwen (control) groups. To control for confounding factors, we used conditional logistic regression in a propensity-score matched (PSM) cohort (1 : 1 balanced), as well as logistic regression without matching as sensitivity analysis. A total of 4918 patients were included, 2760 of whom received Lianhua Qingwen capsules and 2158 of whom did not. In the PSM model, after adjusting for confounders, the in-hospital mortality was similar between the Lianhua Qingwen group and the control group (6.8% vs. 3.3%, adjusted OR, 0.66 [95% CI, 0.38-1.15], *p* = 0.138). The negative conversion rate of Severe Acute Respiratory Syndrome Coronavirus-2 (SARS-CoV-2) infection was higher in the Lianhua Qingwen group (88.3% vs. 96.1%, adjusted OR, 4.02 [95% CI, 2.58-6.25], *p* < 0.001). The incidence of acute liver injury was comparable between the two groups (14.0% vs. 11.5%, adjusted OR: 0.85 [95% CI, 0.71-1.02], *p* = 0.083), and the incidence of acute kidney injury was lower in the Lianhua Qingwen group (5.3% vs. 3.0%, adjusted OR: 0.71 [95% CI, 0.50-1.00], *p* = 0.048). Treatment with Lianhua Qingwen capsules was not significantly associated with in-hospital mortality in COVID-19 patients. In the Lianhua Qingwen group, the negative conversion rate of SARS-CoV-2 infection was higher and the incidence of acute kidney injury was lower than in the control group.

## 1. Introduction

While the Coronavirus Disease 2019 (COVID-19) pandemic has been ongoing for nearly three years, the disease epidemic has been moderated in most countries due to the successful development and administration of vaccines. However, severe conditions and death still occur among some people infected with Severe Acute Respiratory Syndrome Coronavirus-2 (SARS-CoV-2), and effective therapeutic drugs remain scarce [1–4]. Lianhua Qingwen is a traditional Chinese medicine (TCM) with botanical ingredients such as forsythia, honeysuckle, ephedra, bitter almond, and others [5]. It is processed into capsules and granules and was first marketed in China in 2004 to prevent and treat viral infections of the respiratory tract [6].

As early as the 2020 pandemic, Lianhua Qingwen capsules and granules were recommended as therapeutic agents in six successive versions of the diagnosis and treatment guidelines for COVID-19 [4, 7]. On April 14, 2020, the indication that “in the routine treatment of COVID-19, it can be used for fever, cough, and malaise caused by a mild or moderate type of disease” was approved to be added to the usage of Lianhua Qingwen capsules by the National Medical Products Administration of the People's Republic of China [8]. Several studies have shown the efficacy and safety of Lianhua Qingwen for the treatment of COVID-19. However, the number of cases in these studies was small, which provided insufficient strength of the evidence supporting the effectiveness of Lianhua Qingwen for COVID-19 [8–10].

The extensive use of Lianhua Qingwen for COVID-19 treatment in the Chinese population underscores the urgent need for a comprehensive assessment of its safety and efficacy. Utilizing the COVID-19 database from early 2020, we conducted a retrospective study on the effectiveness and safety of Lianhua Qingwen to provide evidence for the appropriate use of this treatment for COVID-19.

## 2. Materials and Methods

### 2.1. Study Design

This multicenter retrospective study was conducted between December 19, 2019 and April 26, 2020 at four hospitals in Wuhan, China. Based on the rapid advice guideline for the diagnosis and management of COVID-19, data on patients with a confirmed diagnosis of COVID-19 were collected [4]. This study was approved by the Medical Ethics Committee of the Zhongnan Hospital of Wuhan University.

**Figure 1:**
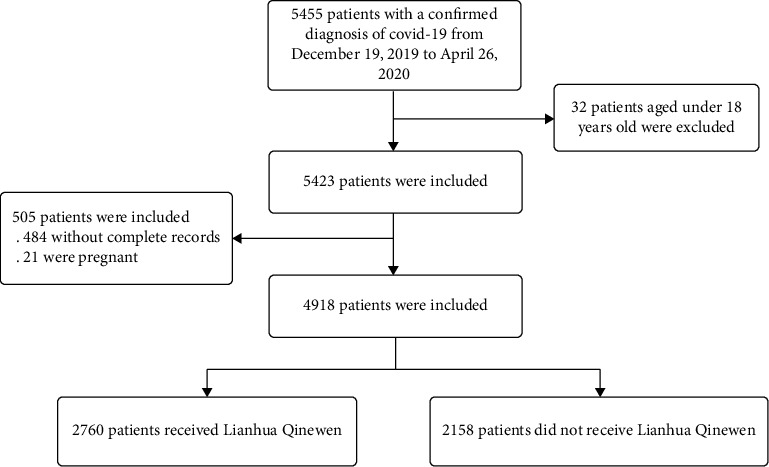
Flowchart of inclusion criteria.

After excluding 32 patients aged <18 years, 484 patients without complete records, and 21 pregnant patients, a total of 4918 patients were included in the analysis (Figure 1).

### 2.2. Outcomes

The primary outcome in our analysis was effectiveness of Lianhua Qingwen including in-hospital mortality and negative conversion rate of SARS-CoV-2 infection which was conducted in terms of progressive disease and was defined as two consecutive negative nucleic acid tests for at least 24 h between samples from respiratory specimens [4].

The secondary outcome was the safety of Lianhua Qingwen including the incidence of acute liver injury (ALI) and acute kidney injury (AKI). ALI was defined as either (1) alanine aminotransferase (ALT) or aspartate aminotransferase (AST) ≥3 upper limit of normal (ULN); (2) alkaline phosphatase (ALP), total bilirubin (TBIL), or gamma-glutamyl transferase (GGT) ≥2ULN [11, 12]. AKI was defined as one of the following: (1) increase in serum creatinine (SCR) by ≥26.5 (*μ*mol/L) within 48 hours, (2) increase in SCR ≥1.5 times baseline within the previous 7 days, or (3) urine volume ≤0.5 ml/kg/h for 6 hours [12].

### 2.3. Data Extraction and Management

Clinical data on patient characteristics, treatments during hospitalization, and outcomes were extracted from the hospital electronic medical record database consisting of front-pages, progress notes, laboratory testing results, and medication administration records. We included the following covariates: age (years), sex (female or male), time from symptom onset to admission (days), severity at admission (nonsevere group: mild or moderate cases; severe group: severe or critically severe cases) according to the Chinese management guidelines for COVID-19 (six versions), symptoms at admission (fever, cough, dyspnea, fatigue, diarrhea, or vomiting, yes or no), and comorbidities (diabetes, hypertension, cancer, and heart diseases, yes or no). In addition, the drugs (antiviral, adrenocortical hormone, or anticoagulant therapy) used were considered.

### 2.4. Statistical Analysis

Categorical variables were described as counts and percentages, and continuous variables as means ± standard deviations or medians with interquartile ranges (IQRs). Chi-square (*χ*^2^) or Fisher's exact tests were used to compare categorical variables. The Mann–Whitney *U* test or *t*-test was used for continuous variables. Propensity score matching (PSM) was used to adjust for confounding factors and reduce the bias. A propensity score refers to the probability that a patient would be assigned to a certain intervention, given a set of covariates [13].

Patients who did or did not use Lianhua Qingwen were matched 1 : 1 based on similar or identical propensity scores. PSM was achieved using the MatchIt package in R using greedy nearest neighbor matching (maximum caliper distance = 0.1). In our analysis, only variables such as age, sex, time from symptom onset to admission, severity and symptoms at admission, and comorbidities were included in multivariable logistic regression to calculate propensity scores. Equivalence between the two groups was examined using the methods described above for categorical and continuous variables. Then, conditional logistic regressions with or without drug adjustment were used to explore the associations between Lianhua Qingwen capsules and outcomes.

Furthermore, the results of logistic regression based on an unmatched cohort were also compared with PSM [14, 15]. In the logistic regression, the strategies of the adjusted covariates were similar to those used in PSM. Statistical significance was defined as a two-tailed *p* value <0.05.

## 3. Results

### 3.1. Baseline Characteristics

A total of 4918 COVID-19 patients were enrolled.  Table 1 shows the patients' characteristics upon admission. Treatment with Lianhua Qingwen capsules was provided to 2760 patients (56.1%) and was not provided to 2158 patients (43.9%). Lianhua Qingwen capsules of 1.4 g were orally administrated three times a day, and the median duration of Lianhua Qingwen treatment was 10 days (IQR: [3–16]). Of them, the median age was 61 years (IQR: [49-69]), and 48.3% (2377) of patients were women. Of the patients, 43.7% (2147) had severe condition at admission, 16.2% (796) had diabetes, 29.1% (1430) had hypertension, 0.9% (45) had cancer, and 6.2% (306) had heart diseases. Patients who received Lianhua Qingwen were older (59 (IQR: [47-68]) vs. 62 (IQR: [51-69]), *p* < 0.001) and spent less time from symptom onset to admission (16 (IQR: [8-30]) vs. 13 (IQR: [7-20]), *p* < 0.001). Compared with control group, Lianhua Qingwen users were easier to have symptoms of fever (51.4% vs. 59.9%, *p* < 0.001), cough (35.4% vs. 43.7%, *p* < 0.001), and fatigue (23.2% vs. 28.2%, *p* < 0.001). We also found that antiviral therapy was more frequently used in the Lianhua Qingwen group (63.9% vs. 87.0%, *p* < 0.001), whereas anticoagulant therapy was used less frequently in the Lianhua Qingwen group (16.2% vs. 11.1%, *p* < 0.001) (Table 1).

### 3.2. Primary Outcomes

**Figure 2:**
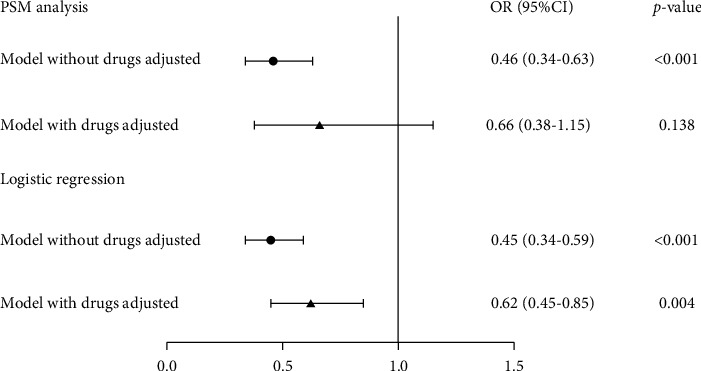
Association between Lianhua Qingwen use and in-hospital mortality.

A total of 1997 participants from the Lianhua Qingwen group were 1 : 1 matched to the 1997 participants from the control group. Their characteristics were well-balanced (Table S1). In PSM analysis adjusted for age, sex, time from symptom onset to admission, severity and symptoms at admission, and comorbidities, Lianhua Qingwen decreased in-hospital mortality in COVID-19 patients (adjusted OR, 0.46 [95% CI, 0.34-0.63], *p* < 0.001). After further adjusting drug use (antiviral, adrenocortical hormone, and anticoagulant therapy), Lianhua Qingwen was not significantly associated with in-hospital mortality (adjusted OR, 0.66 [95% CI, 0.38-1.15], *p*=0.138). In a logistic model, the use of Lianhua Qingwen was associated with a lower risk of in-hospital mortality in COVID-19 patients after adjusting for age, sex, time from symptom onset to admission, severity and symptoms at admission, and comorbidities (adjusted OR, 0.45 [95% CI, 0.34-0.59], *p* < 0.001). After further adjustment for drug use, Lianhua Qingwen was also associated with a declined in-hospital mortality in COVID-19 patients (adjusted OR, 0.62 [95% CI, 0.45-0.85], *p*=0.004) (Figure 2). The Kaplan–Meier curve for survival probability by Lianhua Qingwen use from the day of COVID-19 diagnosis and continued for 21 days or until death in PSM analysis is shown in Figure S1.

Subsequently, after excluding 1804 patients without SARS-Cov-2 RNA date, 117 first used Lianhua Qingwen after SARS-CoV-2 RNA returned to negative, and 2997 patients were included for negative conversion rate of COVID-19 RNA. A total of 1607 patients used Lianhua Qingwen, and 1390 patients were in the control group (Figure S2). Patients who received Lianhua Qingwen were older (57.0 (IQR: [45.3-66.0]) vs. 61.0 (IQR: [50.0-69.0]), *p* < 0.001) and spent less time from symptom onset to admission (20.0 (IQR: [7.0-30.0]) vs. 14.0 (IQR: [7.0-22.0]), *p* < 0.001). Compared with control group, Lianhua Qingwen users were easier to have fever (48.7% vs. 56.4%, *p* < 0.001), cough (48.3% vs. 62.6%, *p* < 0.001), and fatigue symptoms (30.2% vs. 39.3%, *p* < 0.001). We also found that antiviral therapy was more frequently used in the Lianhua Qingwen group (53.6% vs. 83.1%, *p* < 0.001), whereas anticoagulant therapy was used less frequently in the Lianhua Qingwen group patients (14.2% vs. 9.3%, *p* < 0.001) (Table S2).

A total of 1188 participants who used Lianhua Qingwen were matched with 1188 participants from the control group. The characteristics of the two groups were well-balanced (Table S3). In PSM model adjusted for age, sex, time from symptom onset to admission, severity and symptoms at admission, and comorbidities, Lianhua Qingwen treatment was associated with an elevated negative conversion rate of SARS-CoV-2 (88.3% vs. 96.1%, adjusted OR, 3.21 [95% CI, 2.27-4.54], *p* < 0.001) (Figure S3). After further adjustment for drug use, the use of Lianhua Qingwen was also significantly associated with an increased negative conversion rate of SARS-CoV-2 (adjusted OR, 4.02 [95% CI, 2.58-6.25], *p* < 0.001) (Figure S3). Similarly, in a logistic model with age, sex, time from symptom onset to admission, severity and symptoms at admission, and comorbidities, we also found an association between Lianhua Qingwen treatment and an increased negative conversion rate of SARS-CoV-2 (adjusted OR, 3.56 [95% CI, 2.60-4.92], *p* < 0.001). After further adjustment for drug use, Lianhua Qingwen was also associated with an increased negative conversion rate of SARS-CoV-2 (adjusted OR, 3.86 [95% CI, 2.79-5.38], *p* < 0.001) (Figure S3).

### 3.3. Secondary Outcomes

COVID-19 patients who received Lianhua Qingwen had a comparable incidence of ALI (crude rate, 14.0% [95% CI, 12.5%-15.5%] vs. 11.5% [95% CI, 10.3%-12.8%], adjusted OR: 0.85 [95% CI, 0.71-1.02], *p* = 0.083) and a slightly lower incidence of AKI (crude rate, 5.3% [95% CI, 4.4%–6.4%] vs. 3.0% [95% CI, 2.4%–3.8%]; adjusted OR: 0.71 [95% CI, 0.50–1.00], *p* = 0.048) compared with the patients who did not receive Lianhua Qingwen after adjusting for age, sex, time from symptom onset to admission, severity and symptoms at admission, and comorbidities as well as antiviral, adrenocortical hormone, and anticoagulant therapy (Table 2).

## 4. Discussion

The treatment of COVID-19 remains a pressing issue during the current worldwide pandemic. Although several meta-analyses and systematic reviews have been published on this topic [16, 17], our work represents the first retrospective study examining the effectiveness and safety of the Lianhua Qingwen capsules in a comparatively large number of in-hospital COVID-19 patients from multicenters. Although Lianhua Qingwen treatment was not associated with in-hospital mortality in the present study, the results still revealed that Lianhua Qingwen capsules effectively improved the negative conversion rate of SARS-CoV-2 infection and reduced the incidence of AKI in COVID-19 patients.

In China, TCM has a long history of treating against influenza [18]. Lianhua Qingwen capsule is a Chinese patent medicine composed of eleven herbs. Previous studies on TCM therapy and modern pharmaceuticals have proven that the ingredients in Lianhua Qingwen capsules, such as isatis root, forsythia, herbal houttuynia, and honeysuckle, have a very significant effect on viral respiratory diseases such as SARS, influenza (including H1N1 and H7N9), chronic rhinosinusitis, tonsillitis, and hand-foot-and-mouth disease by relieving the symptoms of fever, headache, dizziness, fatigue, and rhinorrhea [5, 6, 19]. Wu et al. have reported that the combination of Lianhua Qingwen granules and peramivir sodium chloride injection could shorten the progress of the disease and improve C-reactive protein (CRP), procalcitonin (PCT) levels, and interleukin (IL)-6 levels with promising potency in influenza patients [20]. Based on previous clinical experiences, Lianhua Qingwen has been recommended to treat patients with COVID-19 after the pandemic outbreak. Fan et al. have reported that Lianhua Qingwen significantly alleviated the symptoms of respiratory infection in 66 patients with COVID-19 on the basis of routine treatment [9]. Li et al. suggested that a combination therapy consisting of Lianhua Qingwen, umifenovir, ribavirin, and lopinavir/ritonavir could be an optional treatment approach for 151 severe patients with COVID-19 [21].

In our study, Lianhua Qingwen did not appear to affect in-hospital mortality; however, it significantly increased the negative conversion rate of SARS-CoV-2 infection. It has been reported that the active component of Lianhua Qingwen may inhibit viral replication by blocking the binding of SARS-CoV-2 to angiotensin-converting enzyme 2 (ACE2) [22] and could reduce the virions in infected cells, as well as change the surface virions of infected cells conducted by Nanshan Zhong et al.[23]. Meanwhile, research has shown that Lonicera japonica and Forsythia (main components of Lianhua Qingwen capsules) can inhibit the binding of the novel coronavirus to ACE2 [24]. Similarly, Rheum palmatum (another main component of Lianhua Qingwen capsules) can effectively block the interaction between S protein and ACE2 [25]. A network pharmacology analysis suggested that Lianhua Qingwen capsules might potentially treat and prevent COVID-19 by targeting the serine/threonine protein kinase (Akt1) [26], which is involved in viral infection, lung injury, and lung fibrosis [27]. The impact of Lianhua Qingwen capsules on increasing the negative conversion rate of SARS-CoV-2 in our study is supported by the above mechanisms.

Although COVID‐19 was first found to target to the respiratory system, growing evidence highlights the wide distribution of ACE2 enables SARS-CoV-2 to cause a systemic disease characterized by multiple organ involvement [28, 29]. The liver and kidney may be among the target organs of SARS-COV-2 [30, 31]. In our study, we found that the incidence of ALI was comparable between Lianhua Qingwen and the control groups. Notably, we first observed a lower risk of AKI in COVID-19 patients taking Lianhua Qingwen capsules. Early recognition and application of preventive and therapeutic measures to limit successive AKI are essential to reduce mortality in COVID-19 patients. Therefore, relevant research is required to investigate the underlying mechanisms.

To this study, there are still potential limitations. First, the sample was limited to a restricted number of hospitalized patients who were crudely classified into Lianhua Qingwen and non-Lianhua Qingwen groups. It is also possible that the comparatively limited sample size in each group led to a lack of power with an increased beta risk. Second, although we used PSM to control for important characteristics, information not included in the analysis may lead to potential confounders. Third, the protocol design of our study was different from that of a randomized control trial. The association between Lianhua Qingwen and clinical outcomes should continue to be clarified through high-quality clinical trials.

## 5. Conclusions

Our study demonstrated that treatment with Lianhua Qingwen capsules was not associated with in-hospital mortality, but it increased the negative conversion rate of SARS-CoV-2 infection and reduced the incidence of AKI in COVID-19 patients, suggesting that Lianhua Qingwen combined with other therapeutic drugs may be a promising strategy for COVID-19.

## Figures and Tables

**Table 1:** Baseline characteristics of COVID-19 patients by Lianhua Qingwen use.

Characteristic	Overall (*N* = 4918)	Control (*N* = 2158)	LHQW (*N* = 2760)	*p-*Value^*∗*^
Age (years)	61.0 (49.0-69.0)	59.0 (47.0-68.0)	62.0 (51.0-69.0)	**<0.001**

Gender (%)				0.054
Female	2377 (48.3)	1077 (49.9)	1300 (47.1)	
Male	2541 (51.7)	1081 (50.1)	1460 (52.9)	

Time from symptom onset to admission (days)	14.0 (7.0-27.0)	16.0 (8.0-30.0)	13.0 (7.0-20.0)	**<0.001**
Severity at admission (*n*, %)				0.138
Nonsevere^a^	2771 (56.3)	1242 (57.6)	1529 (55.4)	
Severe^b^	2147 (43.7)	916 (42.4)	1231 (44.6)	

Symptoms at admission (*n*, %)				
Fever	2763 (56.2)	1110 (51.4)	1653 (59.9)	**<0.001**
Cough	1969 (40.0)	764 (35.4)	1205 (43.7)	**<0.001**
Dyspnea	1279 (26.0)	532 (24.7)	747 (27.1)	0.060
Fatigue	1278 (26.0)	500 (23.2)	778 (28.2)	**<0.001**
Diarrhea or vomiting	1059 (21.5)	457 (21.2)	602 (21.8)	0.615

Comorbidities (*n*, %)				
Diabetes	796 (16.2)	370 (17.1)	426 (15.4)	0.115
Hypertension	1430 (29.1)	601 (27.8)	829 (30.0)	0.100
Cancer	45 (0.9)	20 (0.9)	25 (0.9)	1.000
Heart diseases	306 (6.2)	133 (6.2)	173 (6.3)	0.927

Medication (*n*, %)				
Antiviral	3778 (76.8)	1378 (63.9)	2400 (87.0)	**<0.001**
Adrenocortical hormone	1447 (29.4)	666 (30.9)	781 (28.3)	0.054
Anticoagulant	657 (13.4)	350 (16.2)	307 (11.1)	**<0.001**

LHQW, Lianhua Qingwen capsules; ^a^including mild and moderate cases. ^b^including severe and critical cases.

**Table 2:** Associations between Lianhua Qingwen and safety outcomes.

Safety outcome	Crude rate (95% CI, %)	Unadjusted		Adjusted^*∗*^	
		OR (95% CI)	*p*-Value	OR (95% CI)	*p*-Value
Acute liver injury					
Control	**14.0 (12.5-15.5)**	1 (ref)		1 (ref)	
LHQW	**11.5 (10.3-12.8)**	**0.80 (0.68-0.95)**	**0.010**	**0.85 (0.71-1.02)**	**0.083**

Acute kidney injury					
Control	**5.3 (4.4-6.4)**	1 (ref)		1 (ref)	
LHQW	**3.0 (2.4-3.8)**	**0.56 (0.42-0.74)**	**<0.001**	**0.71 (0.50-1.00)**	**0.048**

LHQW, Lianhua Qingwen capsules; OR, odds ratio; Cl, confidence interval, and ref, reference. ^*∗*^Association was adjusted for age, sex, time from symptom onset to admission, severity and symptoms at admission, comorbidities, and antiviral, adrenocortical hormone, and anticoagulant therapy.

## Data Availability

The original data are available from the corresponding author upon request.

## Supplementary Materials

Supplementary Materials Figure S1. Kaplan–Meier survival curves for in-hospital mortality by Lianhua Qingwen use. Figure S2. Flowchart of the analysis of negative conversion rate. Figure S3. Association of Lianhua Qingwen use with negative conversion rate. Table S1. Baseline characteristics of COVID-19 patients by Lianhua Qingwen use after propensity score matching. Table S2. Baseline characteristics of COVID-19 patients by Lianhua Qingwen in the analysis of negative conversion rate. Table S3. Baseline characteristics of patients with Lianhua Qingwen in the analysis of negative conversion rate after propensity score matching.
