# Supplementary material for: Effectiveness and Safety of Lianhua Qingwen Capsules for COVID-19: A Propensity-Score Matched Cohort Study
Source: Evid Based Complement Alternat Med. 2023 Feb 17;2023:6028554. doi: 10.1155/2023/6028554 (PMC9957644; doi:10.1155/2023/6028554)
Supplement: Supplementary Materials — Figure S1. Kaplan–Meier survival curves for in-hospital mortality by Lianhua Qingwen use. Figure S2. Flowchart of the analysis of negative conversion rate. Figure S3. Association of Lianhua Qingwen use with negative conversion rate. Table S1. Baseline characteristics of COVID-19 patients by Lianhua Qingwen use after propensity score matching. Table S2. Baseline characteristics of COVID-19 patients by Lianhua Qingwen in the analysis of negative conversion rate. Table S3. Baseline characteristics of patients with Lianhua Qingwen in the analysis of negative conversion rate after propensity score matching. [file 6028554.f1.docx]

**Figure S1. Kaplan-Meier survival curves for in-hospital mortality by Lianhua Qingwen use**


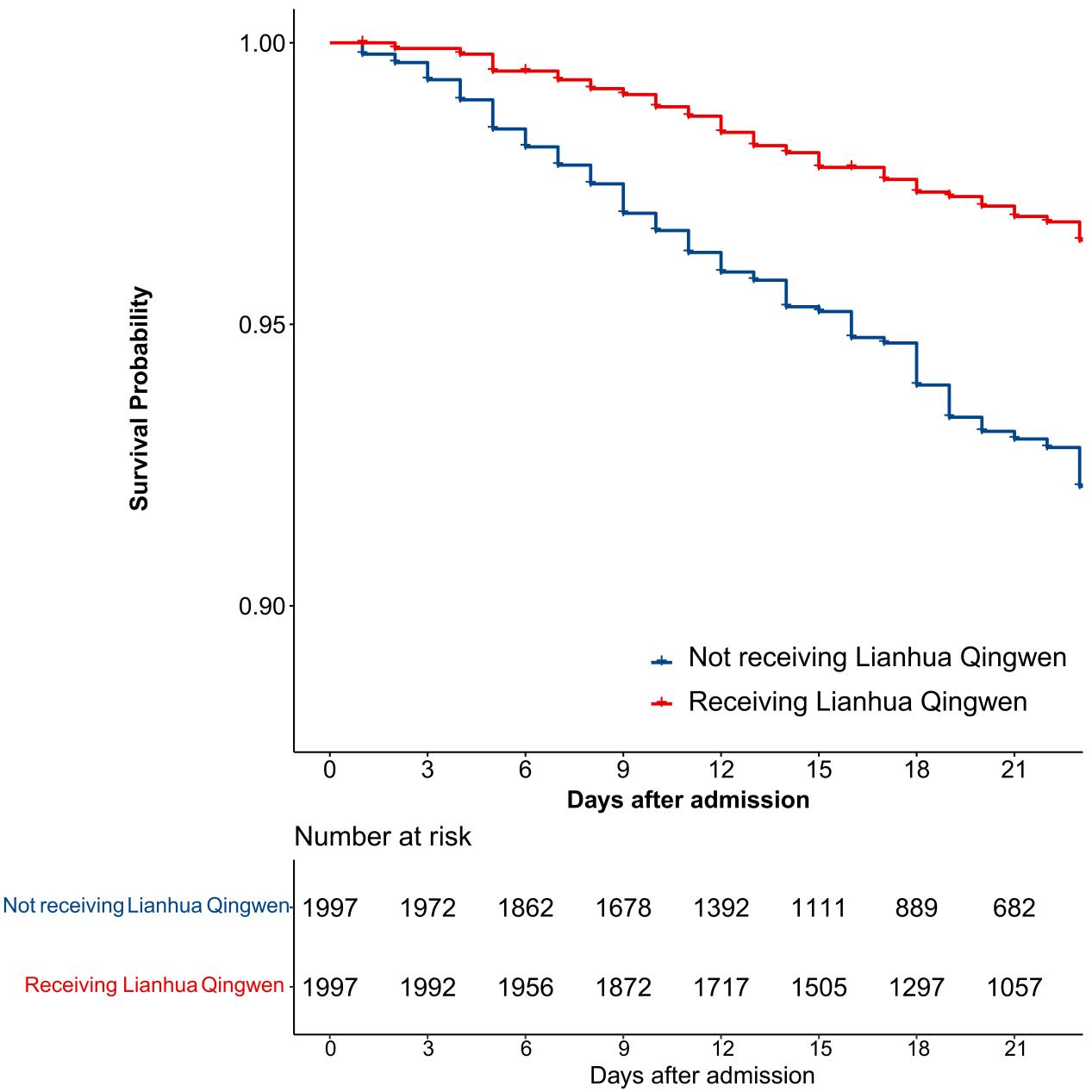


**Figure S2. Flowchart of the analysis of negative conversion rate**


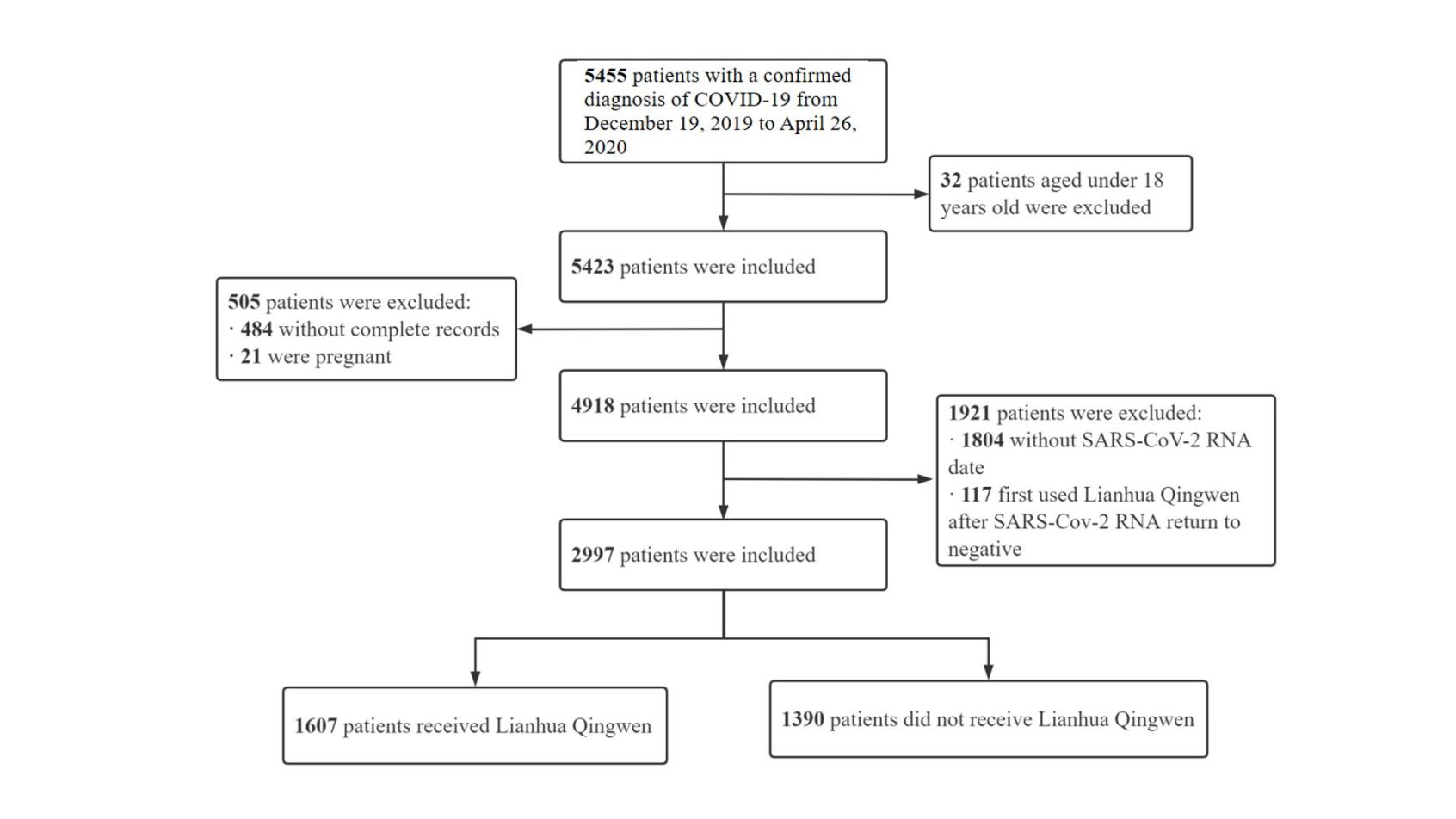


**Figure S3. Association of Lianhua Qingwen use with negative conversion rate**


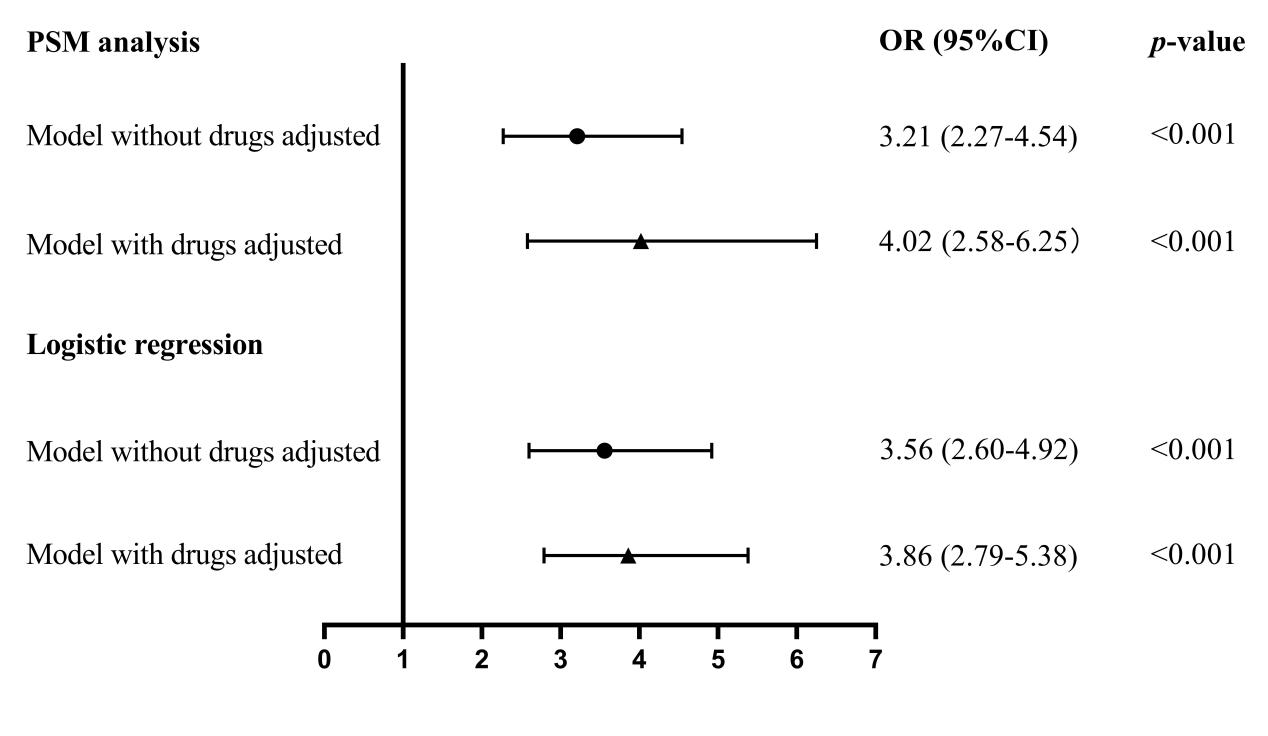


**Table S1. Baseline characteristics of COVID-19 patients by Lianhua Qingwen use after** **propensity score matching**

| **Characteristics** | **Overall**  **(N=3994)** | **Control**  **(N=1997)** | **LHQW**  **(N=1997)** | ***p-*value**^*^ |
| --- | --- | --- | --- | --- |
| Age (years) | 60.0 (49.0-69.0) | 60.0 (48.0-69.0) | 61.0 (49.0-69.0) | 0.407 |
| Gender (%) |  |  |  | 0.393 |
| Male | 1988 (49.8) | 980 (49.1) | 1008 (50.5) |  |
| Female | 2006 (50.2) | 1017 (50.9) | 989 (49.5) |  |
| Time from symptom onset to admission (days) | 15.0 (8.0-28.0) | 15.0 (7.0-30.0) | 15.0 (8.0-24.0) | 0.455 |
| Severity at admission (n,%) | | | | 0.445 |
| Non-severe^a^ | 2219 (55.6) | 1122 (56.2) | 1097 (54.9) |  |
| Severe^b^ | 1775 (44.4) | 875 (43.8) | 900 (45.1) |  |
| Symptoms at admission (n,%) | | | | |
| Fever | 2159 (54.1) | 1065 (53.3) | 1094 (54.8) | 0.374 |
| Cough | 1507 (37.7) | 743 (37.2) | 764 (38.3) | 0.514 |
| Dyspnea | 1026 (25.7) | 509 (25.5) | 517 (25.9) | 0.800 |
| Fatigue | 981 (24.6) | 486 (24.3) | 495 (24.8) | 0.769 |
| Diarrhoea or vomiting | 883 (22.1) | 435 (21.8) | 448 (22.4) | 0.647 |
| Comorbidities (n,%) | | | | |
| Diabetes | 676 (16.9) | 340 (17.0) | 336 (16.8) | 0.899 |
| Hypertension | 1119 (28.0) | 560 (28.0) | 559 (28.0) | 1.000 |
| Cancer | 35 (0.9) | 16 (0.8) | 19 (1.0) | 0.734 |
| Heart diseases | 245 (6.1) | 121 (6.1) | 124 (6.2) | 0.895 |

LHQW, Lianhua Qingwen capsule; ^*^P-values were calculated using the Chi-square test, Fisher’s exact method, or Kruskal-Wallis test. ^a^Including mild and moderate cases. ^b^Including severe and critical cases.

**Table S2. Baseline characteristics of COVID-19 patients by Lianhua Qingwen in the analysis of negative conversion rate**

| **Characteristics** | **Overall**  **(N=2997)** | **Control**  **(N=1390)** | **LHQW**  **(N=1607)** | ***p-*value**^*^ |
| --- | --- | --- | --- | --- |
| Age (years) | 59.0 (48.0-68.0) | 57.0 (45.3-66.0) | 61.0 (50.0-69.0) | **<0.001** |
| Gender (%) |  |  |  | 0.576 |
| Female | 1412 (47.1) | 663 (47.7) | 749 (46.6) |  |
| Male | 1585 (52.9) | 727 (52.3) | 858 (53.4) |  |
| Time from symptom onset to admission (days) | 15.0 (7.0-30.0) | 20.0 (7.0-30.0) | 14.0 (7.0-22.0) | **<0.001** |
| Severity at Admission (n,%) | | | | 0.374 |
| Non-severe^a^ | 2587 (86.3) | 1191 (85.7) | 1396 (86.9) |  |
| Severe^b^ | 410 (13.7) | 199 (14.3) | 211 (13.1) |  |
| Symptoms at admission (n,%) | | | | |
| Fever | 1584 (52.9) | 677 (48.7) | 907 (56.4) | **<0.001** |
| Cough | 1677 (56.0) | 671 (48.3) | 1006 (62.6) | **<0.001** |
| Dyspnea | 565 (18.9) | 235 (16.9) | 330 (20.5) | 0.013 |
| Fatigue | 1051 (35.1) | 420 (30.2) | 631 (39.3) | **<0.001** |
| Diarrhoea or vomiting | 204 (6.8) | 93 (6.7) | 111 (6.9) | 0.871 |
| Comorbidities (n,%) | | | | |
| Diabetes | 361 (12.0) | 180 (12.9) | 181 (11.3) | 0.174 |
| Hypertension | 885 (29.5) | 398 (28.6) | 487 (30.3) | 0.337 |
| Cancer | 27 (0.9) | 11 (0.8) | 16 (1.0) | 0.692 |
| Heart diseases | 186 (6.2) | 89 (6.4) | 97 (6.0) | 0.735 |
| Medication (n,%) | | | | |
| Antiviral | 2080 (69.4) | 745 (53.6) | 1335 (83.1) | **<0.001** |
| Adrenocortical hormone | 771 (25.7) | 322 (23.2) | 449 (27.9) | 0.003 |
| Anticoagulant | 347 (11.6) | 198 (14.2) | 149 (9.3) | **<0.001** |

LHQW, Lianhua Qingwen capsule; ^*^P-values were calculated using the Chi-square test, Fisher’s exact method, or Kruskal-Wallis test. ^a^Including mild and moderate cases. ^b^Including severe and critical cases.

**Table S3. Baseline characteristics of patients with Lianhua Qingwen in the analysis of negative conversion rate after propensity score matching**

| **Characteristics** | **Overall**  **(N=2376)** | **Control**  **(N=1188)** | **LHQW**  **(N=1188)** | ***p-*value**^*^ |
| --- | --- | --- | --- | --- |
| Age (years) | 58.0 (47.0-68.0) | 58.0 (47.0-67.0) | 58.0 (47.0-68.0) | 0.725 |
| Gender (%) |  |  |  | 0.565 |
| Male | 1099 (46.3) | 557 (46.9) | 542 (45.6) |  |
| Female | 1277 (53.7) | 631 (53.1) | 646 (54.4) |  |
| Time from symptom onset to admission (days) | 15.0 (7.0-30.0) | 15.0 (7.0-30.0) | 15.0 (7.0-27.0) | 0.520 |
| Severityas admission (n,%) | | | | 0.724 |
| Non-severe^a^ | 2041 (85.9) | 1024 (86.2) | 1017 (85.6) |  |
| Severe^b^ | 335 (14.1) | 164 (13.8) | 171 (14.4) |  |
| Symptoms at admission (n,%) | | | | |
| Fever | 1224 (51.5) | 608 (51.2) | 616 (51.9) | 0.774 |
| Cough | 1263 (53.2) | 624 (52.5) | 639 (53.8) | 0.565 |
| Dyspnea | 425 (17.9) | 210 (17.7) | 215 (18.1) | 0.830 |
| Fatigue | 797 (33.5) | 390 (32.8) | 407 (34.3) | 0.487 |
| Diarrhoea or vomiting | 169 (7.1) | 86 (7.2) | 83 (7.0) | 0.873 |
| Comorbidities (n,%) | | | | |
| Diabetes | 296 (12.5) | 151 (12.7) | 145 (12.2) | 0.756 |
| Hypertension | 683 (28.7) | 336 (28.3) | 347 (29.2) | 0.650 |
| Cancer | 16 (0.7) | 9 (0.8) | 7 (0.6) | 0.802 |
| Heart diseases | 146 (6.1) | 73 (6.1) | 73 (6.1) | 1.000 |

LHQW, Lianhua Qingwen capsule; ^*^P-values were calculated using the Chi-square test, Fisher’s exact method, or Kruskal-Wallis test. ^a^Including mild and moderate cases. ^b^Including severe and critical cases.
